# Would the number of new cases of hereditary angioedema decrease due to declining fertility rates?

**DOI:** 10.1186/s13223-026-01056-8

**Published:** 2026-08-06

**Authors:** Lauren Wong, Harold Kim, Mark Kuprowski

**Affiliations:** 1 Department of Medicine, Western University, London, ON Canada; 2 Division of Clinical Immunology and Allergy, Department of Medicine, Western University, London, ON Canada

**Keywords:** Hereditary angioedema, Fertility rate, Rare diseases, Pharmaceutical

## Abstract

Hereditary angioedema (HAE) is a rare, potentially life-threatening disorder caused by autosomal dominant mutations in the *SERPING1* gene, leading to deficiency or dysfunction of C1 esterase inhibitor. Recent estimates of global HAE prevalence are 1.22 per 100,000 individuals. With total fertility rates (TFRs) decreasing worldwide, several developed countries are now well below the population replacement rate in an “ultra-low fertility” group. With the decrease in fertility, how rare diseases are approached should be re-examined. In this letter, we describe how continued and sustained declines in total fertility rate and population may influence the future number of new HAE cases. We also address disease-specific factors that may further influence future case count, including reproductive hesitancy related to autosomal dominant inheritance, estrogen-associated exacerbation of angioedema attacks, and potential fertility effects of complement dysregulation. These demographic considerations are relevant to clinicians, researchers, and pharmaceutical companies involved in HAE care. Better characterizing population changes in the context of reduced fertility rates may help inform long-term clinical planning and the development of novel therapies for HAE and other rare diseases.

## Main text

Hereditary angioedema (HAE) is a rare genetic disorder with an autosomal dominant inheritance pattern of mutations in the *SERPING1* gene. HAE type 1, caused by C1 esterase inhibitor deficiency is most common, while type 2 which is due to C1 esterase inhibitor dysfunction accounts for approximately 15% of cases [1]. In this letter, we discuss the potential impact of declining fertility rates on new and total HAE case count.

HAE attacks are characterized by acute localized swelling of the face, extremities, genitals, gastrointestinal and/or respiratory tract [1]. In its most severe form, an attack could result in airway obstruction and asphyxiation, making HAE a potentially fatal disease. Treatments were quite limited, but there has been a significant evolution of treatment options recently, including bradykinin B2 receptor antagonist, plasma-derived C1 esterase inhibitor, plasma kallikrein inhibitor, recombinant C1 esterase inhibitor, and factor XII inhibitor [1]. These medications have led to improvements in angioedema frequency, angioedema severity, potentially life-threatening HAE attacks, and HAE quality of life.

In HAE, there are ongoing trials for continued innovative therapeutic advancements. Pharmaceutical companies are investigating new oral agents and prophylactic treatments with a longer sustained effect for HAE [2]. Studies into gene therapy to deactivate kallikrein production or encode for C1 esterase inhibitor are underway [2]. Drug development is an extensive process that typically takes 12–15 years from initial ideation to market launch, encompassing preclinical testing, clinical trials, regulatory approval, and post-market surveillance [3]. The average total research and development costs for a single drug is 1.07 billion dollars as of 2023 and continues to trend upwards [4]. This does not account for the 90% of drugs that will fail clinical trials [5]. The high costs of drug development translate into high drug prices. This is particularly evident in medications used in rare diseases.

HAE has been diagnosed across the world and there is no clear difference in its distribution across racial or ethnic groups [6]. A recent systematic review and meta-analysis of studies between 2000 and 2024 estimated HAE prevalence to be 1.22 per 100,000, with most having HAE type 1 [7]. Interestingly, the total fertility rate (TFR), defined as the number of children a woman is expected to have over her reproductive life, has been on a gradual decline from 2009 to 2019, with a more sudden drop around the COVID-19 pandemic [8]. Global fertility rates fell from 4.8 in 1970 to 2.2 in 2024 [9]. We are now just above the replacement level of 2.1 children per woman [9]. Fertility rates in the United States reached an all-time low of 1.6 in 2023 [9]. In Canada, the TFR dropped from 1.51 in 2018 to 1.33 in 2022 [8] and 1.25 in 2024, making Canada part of the “ultra-low fertility group” with TFRs below 1.30 [10]. Other countries with such low TFRs include Switzerland, Luxembourg, Finland, Italy, Japan, Singapore, and South Korea [10]. South Korea’s TFR is the lowest in the world at 0.75 in 2024 [11].

**Fig. 1:**
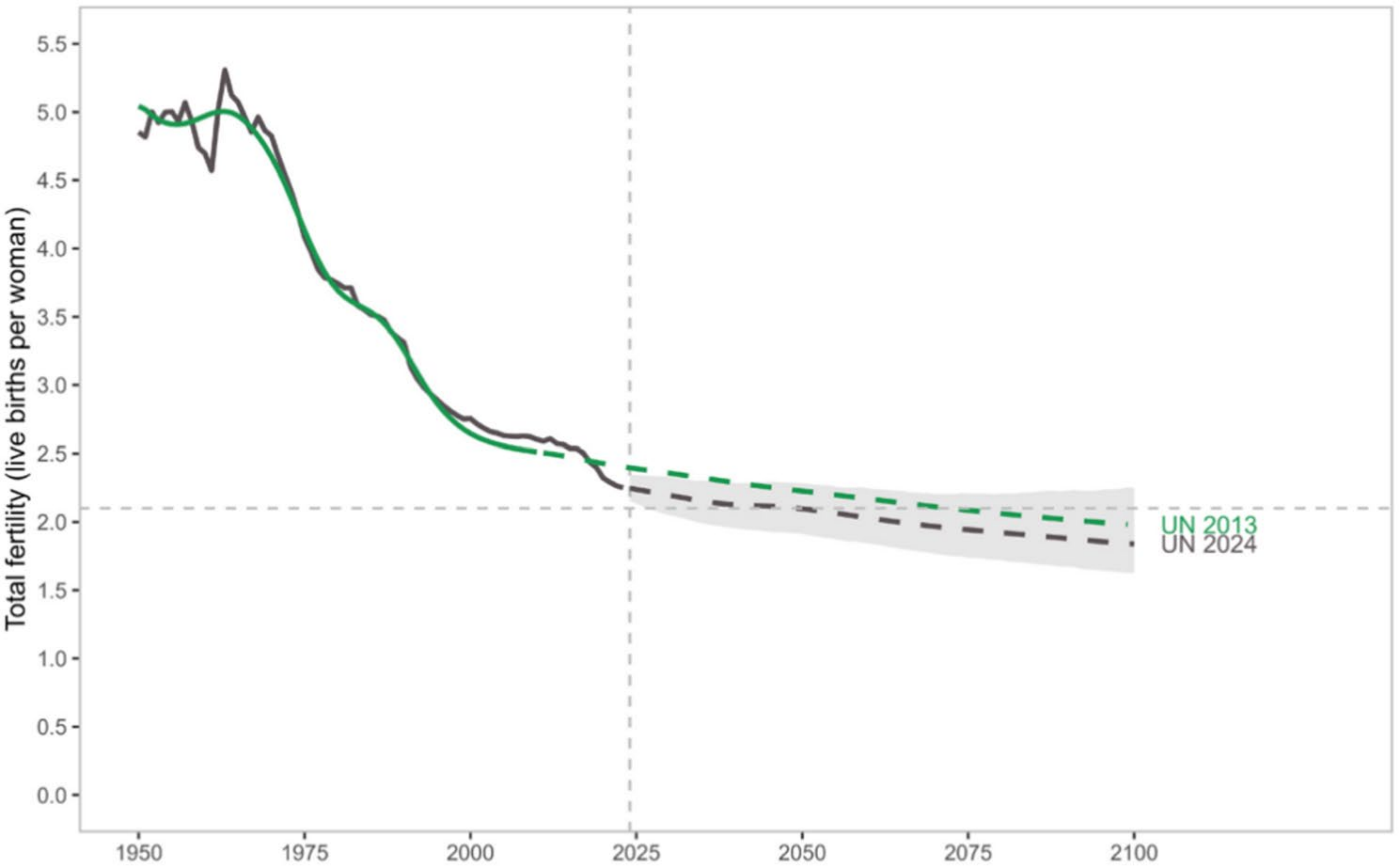
Global total fertility estimates in 2013 and 2024 according to the United Nations, with projections to 2100 [9]

With the widespread reduction in fertility rates across countries, the projected global total fertility rate in 2100 is between 1.6 and 2.2 (Fig. 1), with less than 10% probability that it would surpass the 2.1 replacement rate [9]. Immigration may be able to reduce or offset the declines in national fertility rates seen in developed countries including Canada and the United States, but it is ultimately only a temporary measure. In Canada, during the third quarter of 2025 there was a population drop of 76,000, which is the largest decline seen in Canada since at least the 1940s [12]. While many developed countries are already shrinking or near peak population, continued growth in the developing world, particularly parts of Africa and South Asia delay the global decline [13].

The global patterns of declining fertility rates suggests that the future case count of HAE would be projected to fall. In Canada, for example, with the most recent TFR of 1.25 in comparison to the population replacement rate of 2.1, there would be a 40.48% reduction in the population per generation. One generation is 25–30 years. Therefore, based on the estimated HAE worldwide prevalence of 1.22 in 100,000 [7] and current Canadian population of 41.7 million, there are approximately 509 HAE cases. Theoretically, the number of new HAE cases, assuming prevalence rate remains the same, could decrease by 40.67% per generation. The total number of HAE cases, however, would decrease more slowly as there would still be living HAE patients from previous generations.

We acknowledge that there are limitations to this calculation. The 40.67% does not account for immigration, as mentioned above, and age-related effects where HAE patients now have a longer life expectancy due to better understanding of disease and availability of therapies. Improved awareness and treatment of HAE is predominantly in the developed world though, which is more reliant on immigration to maintain the population. In contrast, countries in the developing world have greater potential for underdiagnosis and inaccessibility to HAE treatments, both of which would contribute to a lower case count. De novo mutations can also affect the total case count, however that should already be accounted for in current incidence and prevalence estimates that are based on real-world data. Ultimately, population modeling is complex and necessary to capture these factors for a rounded assessment.

Another consideration is the fertility of known HAE patients. More than one third of HAE patients are hesitant about having children due to their HAE [14]. Patients report anxiety about the autosomal dominance inheritance pattern of HAE, which carries a risk of passing the disease to the next generation, who may also experience HAE attacks and complications [15]. Increased exposure to estrogen such as in pregnancy is also known to trigger angioedema attacks [16]. While there are no epidemiological studies documenting impaired fertility in HAE patients, there is evidence that active complement in follicular fluid supports the mechanism of ovulation [16]. HAE patients have prominent reductions in C1 inhibitor and classical complement pathway activity compared to healthy women, and often have cystic ovaries, which may affect ovarian function and consequently, fertility [17]. Dysregulation in the complement system has also been implicated in severe obstetrical complications including early pregnancy loss, preeclampsia, and pre-term birth [16]. Prophylactic therapy or comprehensive delivery plans are required to optimize pregnancy in these patients [16]. While the studies in literature are limited at this point due to the small HAE patient population, it can be argued that fertility factors may also contribute to reduced cases of HAE.

True estimates of total HAE cases with the falling fertility rates would inform care for the condition. The predicted reduction in HAE cases offers a new perspective that may cause pharmaceutical companies to reframe their approach to HAE drug development, given the significant time and financial investments associated with each new drug. It is also interesting to consider what implications the decreasing TFR will have for other rare genetic disorders such as hemophilia A and phenylketonuria, for which extensive pharmaceutical studies including gene therapy are also currently underway. Prevalence and incidence modeling based on TFR for rare diseases can be used to help predict new number of cases and should be considered when developing new therapies. Continued advocacy for patients to access existing treatments is also important.

In conclusion, with declining fertility rates globally and with more countries entering the “ultra-low fertility” category, the number of new HAE cases is expected to decrease. Without immigration or increased reproduction to maintain or increase population, especially in the developed world, there may be a reduction in HAE cases even within one generation. While ongoing advancement of HAE therapies is strongly encouraged, these population changes should be considered as new HAE products are developed.

## Data Availability

No datasets were generated or analysed during the current study.

## Abbreviations

HAE: Hereditary angioedema
TFR: Total fertility rate

## References

[1] Longhurst HJ, Bork K. Hereditary angioedema: causes, manifestations and treatment. Br J Hosp Med (Lond). 2016;77(7):391–8. 10.12968/hmed.2006.67.12.22439. 10.12968/hmed.2006.67.12.22439 17328450

[2] Hereditary Angioedema International. HAE treatments in development [Internet]. 2025 [cited 2026 Feb 10]. Available from: https://haei.org/hae-treatments-in-development/

[3] Singh N, Vayer P, Tanwar S, Poyet J-L, Tsaioun K, Villoutreix BO. Drug discovery and development: introduction to the general public and patient groups. Front Drug Discov 3:1201419. 10.3389/fddsv.2023.1201419

[4] Innovation, Science and Economic Development Canada. Pharmaceutical industry profile [Internet]. Ottawa (ON): Government of Canada; 2025 [cited 2026 Feb 10]. Available from: https://ised-isde.canada.ca/site/canadian-life-science-industries/en/biopharmaceuticals-and-pharmaceuticals/pharmaceutical-industry-profile

[5] Sun D, Gao W, Hu H, Zhou S. Why 90% of clinical drug development fails and how to improve it? Acta Pharm Sinica B. 2022;12(7):3049–62. 10.1016/j.apsb.2022.02.002. 10.1016/j.apsb.2022.02.002 PMC9293739 35865092

[6] DiMasi JA, Grabowski HG, Hansen RW. Innovation in the pharmaceutical industry: new estimates of R&D costs. J Health Econ. 2016;47:20–33. 10.1016/j.jhealeco.2016.01.012. 26928437 10.1016/j.jhealeco.2016.01.012

[7] Fisch SA, Rundle AG, Neugut AI, Freedberg DE. Worldwide prevalence of hereditary angioedema: a systematic review and meta-analysis. Int Arch Allergy Immunol. 2025;186(8):802–10. 10.1159/000543321. 39827848 10.1159/000543321

[8] Statistics Canada. Fertility: overview, 2022 [Internet]. Ottawa (ON): Statistics Canada; 2024 [cited 2026 Feb 10]. Available from: https://www150.statcan.gc.ca/n1/pub/91f0015m/91f0015m2024001-eng.htm

[9] United Nations, Department of Economic and Social Affairs, Division P. *World Fertility 2024* [Internet]. New York (NY): United Nations; 2025 [cited 2026 Feb 10]. Available from: https://www.un.org/development/desa/pd/sites/www.un.org.development.desa.pd/files/undesa_pd_2025_wfr_2024_final.pdf

[10] Statistics Canada. Canada’s fertility rate reaches new low in 2024 [Internet]. Ottawa (ON): Statistics Canada; 2025 [cited 2026 Feb 10]. Available from: https://www150.statcan.gc.ca/n1/daily-quotidien/250924/dq250924d-eng.htm

[11] Lee J, Yi H. South Korea’s policy push springs to life as world’s lowest birthrate rises. *Reuters* [Internet]. 2025 Feb 26 [cited 2026 Feb 10]. Available from: https://www.reuters.com/world/asia-pacific/south-koreas-policy-push-springs-life-worlds-lowest-birthrate-rises-2025-02-26/ 26/#:~:text = The%20stakes%20are%20high%2 C%20as,lowest%20in%202023%20at%200.72.

[12] McCarthy S. Canada posts largest population decline since 1940s. *The Globe and Mail* [Internet]. 2025 [cited 2026 Feb 10]. Available from: https://www.theglobeandmail.com/business/article-canada-population-decline-third-quarter-statistics-canada/

[13] United Nations Department of Economic and Social Affairs, Population Division. (2024). World Population Prospects 2024: Summary of Results (UN DESA/POP/2024/TR/NO. 9).

[14] Fijen LM, Petersen RS, Levi M, Lakeman P, Henneman L, Cohn DM. Patient perspectives on reproductive options for hereditary angioedema: a cross-sectional survey study. J Allergy Clin Immunol Pract. 2022;10(9):2483–e24861. 10.1016/j.jaip.2022.05.030. 35690368 10.1016/j.jaip.2022.05.030

[15] Caballero T, Aygören-Pürsün E, Bygum A, Beusterien K, Hautamaki E, Sisic Z et al. The humanistic burden of hereditary angioedema: Results from the burden of illness study in Europe. *Allergy and Asthma Proceedings.* 2014;35(1):47–53. 10.2500/aap.2013.34.3685 10.2500/aap.2013.34.3685 24268449

[16] Triggianese P, Senter R, Petraroli A, Zoli A, Lo Pizzo M, Bignardi D et al. Pregnancy in women with hereditary angioedema due to C1-inhibitor deficiency: RESULTS FROM THE ITACA COHORT study on outcome of mothers and children with in utero exposure to plasma-derived C1-inhibitor. *Frontiers in Medicine*. 2022 Sept 14;9. 10.3389/fmed.2022.930403 10.3389/fmed.2022.930403 PMC9515414 36186797

[17] Perricone, De Carolis, Giacomello, Giacomelli, De Sanctis. Fontana. Impaired human ovarian follicular fluid complement function in hereditary angioedema. Scand J Immunol. 2001;51(1):104–8. 10.1046/j.1365-3083.2000.00652.x. 10.1046/j.1365-3083.2000.00652.x 10632984

